# Supplementary material for: Direct Injection of Recombinant AAV-Containing Solution into the Oviductal Lumen of Pregnant Mice Caused In Situ Infection of Both Preimplantation Embryos and Oviductal Epithelium
Source: Int J Mol Sci. 2022 Apr 28;23(9):4897. doi: 10.3390/ijms23094897 (PMC9105285; doi:10.3390/ijms23094897)
Supplement: Supplementary file 1 [file ijms-23-04897-s001.zip › ijms-1651452-supplementary.pdf]

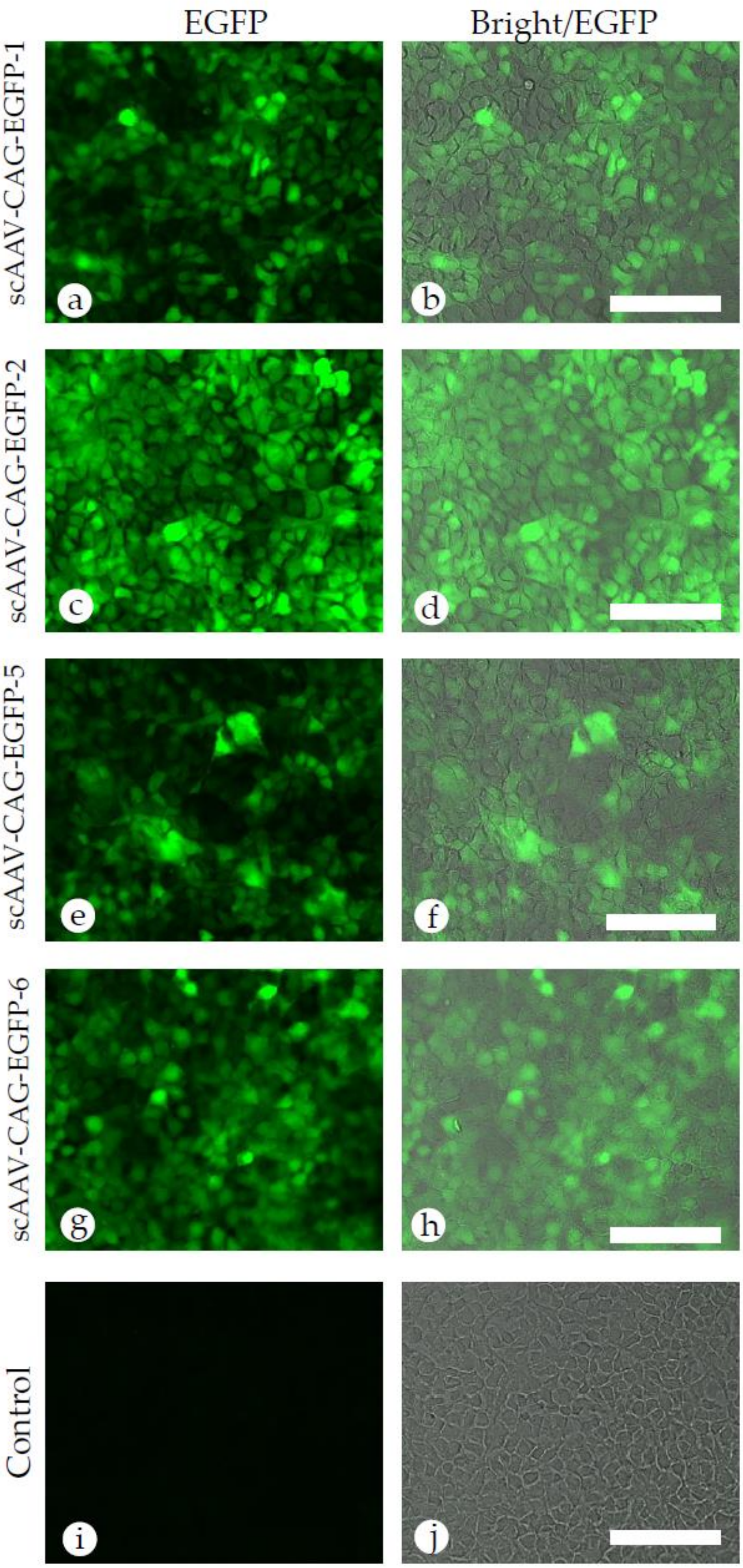

Figure S1. Transduction of EGFP-expressing rAAVs [scAAV-CAG-EGFP-1 (for a and b), -2 (for c and d), -5 (for e and f) and -6 (for g and h)] into HEK293T cells. Control, untreated HEK293T cells (for i and j). Scale bars, 100 μm.

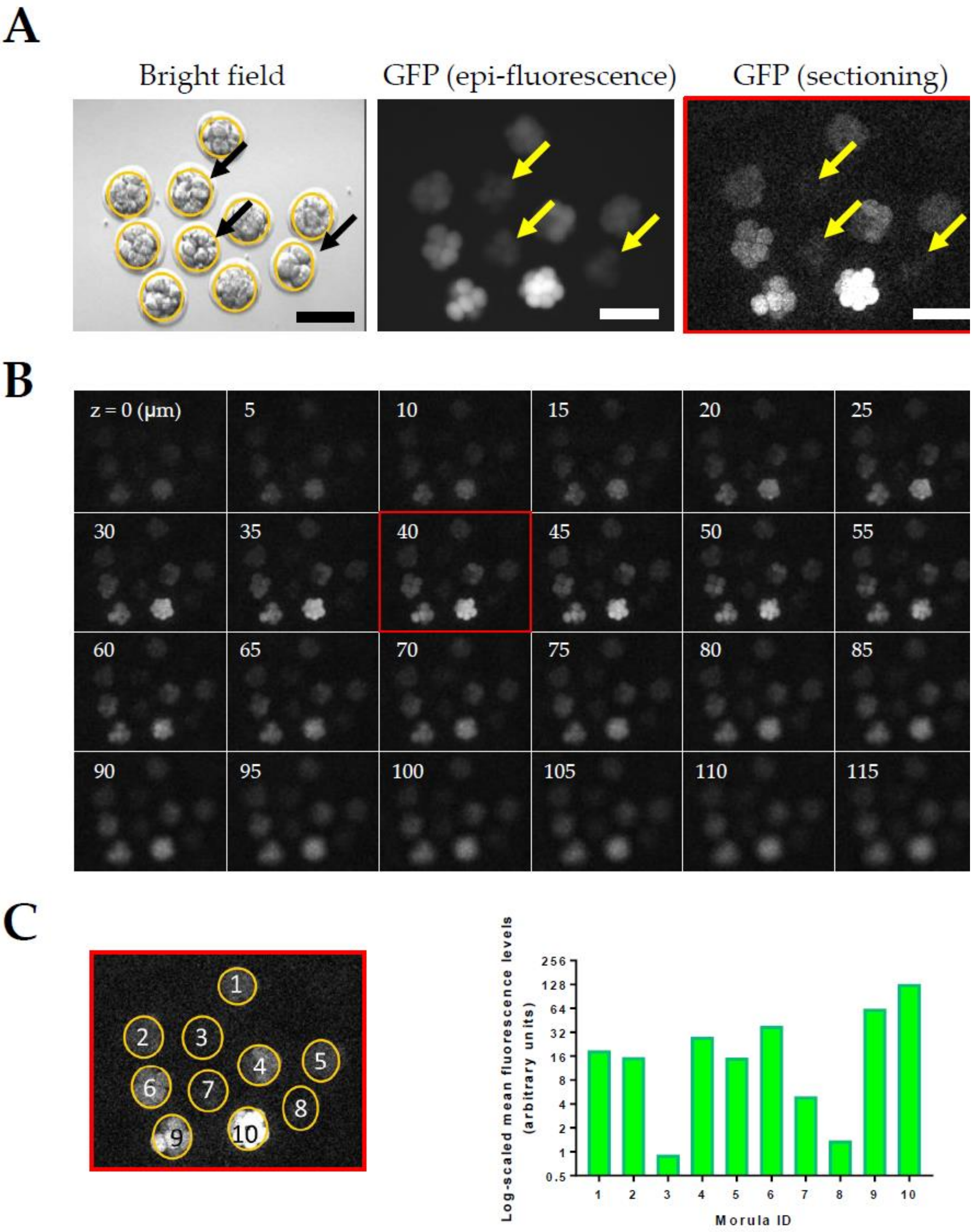

Figure S2. Quantification of GFP fluorescence in optically sectioned images. **A.** The same embryos shown in Figure 1B-a (Bright field) and -b (epi-fluorescence) were compared with an image taken with structured illumination-based optical sectioning (sectioning). Arrows indicate presumable GFP negative cells. B. Serial optical sectioning images of embryos with AAV-based GONAD. The sectioning image in the middle of the stack (red framed) was shown in **A** and **C** as a representative. **C.** Quantification of mean fluorescence levels for each morula in the image shown in the left. Note that morulae #3, #7 and #8 are labeled with arrows in **A**. Scale bars, 100 μm.
